# Response to: Problems and promises of savanna fire regime change

**DOI:** 10.1038/s41467-021-25042-3

**Published:** 2021-08-12

**Authors:** Geoffrey J. Lipsett-Moore, Nicholas H. Wolff, Edward T. Game

**Affiliations:** 1 Cape York Natural Resource Management, Atherton, QLD Australia; 2 The Nature Conservancy, Global Science, Brunswick, ME USA; 3 The Nature Conservancy, Asia Pacific Resource Centre, South Brisbane, QLD Australia

**Keywords:** Climate-change mitigation, Fire ecology

**Replying to** Laris, P. *Nature Communications* 10.1038/s41467-021-25141-1 (2021).

Laris^1^ raises a number of important considerations regarding our analysis of global opportunities for emissions reductions through savanna burning. We agree entirely with Laris on some of the key points: (1) that tens of thousands of local people should be compensated for the ecosystem services they already provide by setting fires early in savanna landscapes, and (2) there is a need to modify fire regimes to reduce late dry season uncontrolled fires in Africa, the majority of emissions identified in our study. However, the question remains, how do we generate sustainable financial incentives to do that? Currently, the only proven mechanism is the savanna burning approach developed in Australia^2,3^, where we can demonstrate previous baselines with high emissions and reduced emissions from changed fire practices (early burning)^2^. We recognise that there are weaknesses in our global analysis when applied at the regional and local scale including: the need for refined emissions factors, refinement of the fire dichotomy, missing small early fires and a history of differing and problematic fire policies across Africa. We used a simple, generic model across sub-Saharan Africa. However, it is likely that climate, rainfall, vegetation, soils and associated emissions factors will vary across sub-Saharan Africa, as suggested by Laris. This will require the development of regional methodologies for Western, Southern, Eastern and Central Africa, with emissions factors and parameters appropriate for the respective regions.

## Emissions trade-off problem

Some of the confusion around the emissions trade-off problem for CH_4_ posed by Laris is one of definition. Laris defines EDS as the period from Dec to Jan immediately after the wet season when fuels are uncured and moisture content is high^4–7^. Under the Australian savanna burning methodology, EDS is defined as the period from 1 Jan to 31 July (inclusive of the wet season) and the LDS as the period from 1 Aug to 31 Dec^2^. In Australia, EDS burning occurs when the grasses have cured and when there is little seasonal difference between EDS and LDS emission factors for CH_4_^8^. The same is also true for southern Africa^9^. If we superimpose the two definitions, then it is evident that EDS in Australia equates to both EDS and Mid Dry Season (MDS) as defined by Laris et al.^10^, where emissions factors are also reduced. Better defining EDS with cured grasses and emissions factors for West Africa may assist in resolving some of these issues.

For those countries with more EDS fires relative to LDS fires and higher emissions from EDS fires—the proposed approach will generate few carbon credits and provide little incentive for improved fire management (e.g. Burkina Faso, Senegal, Benin, Togo and Ghana see Table 1). However, the real opportunity for Africa is likely to be derived from emission avoidance in combination with sequestration across all carbon pools (dead organic matter, living biomass and soils) for the same fire management. In Australia, it has been shown that accumulated dead organic matter sequesters 3.5 times the current emissions avoidance for the same EDS fire management^11^. There is now also an approved combined emissions avoidance and sequestration methodology for Australian savannas^12^. In addition, recent work also indicates that the living biomass component is likely to contribute 3–4 times current emissions avoidance for the same EDS fire management^13^. In many respects, our study^3^, which focused on potential carbon benefits from emissions avoidance only, is an indicator of the greater carbon opportunities that might be gained from all carbon pools for the same EDS fire management^11–14^.

**Table 1:** Mean early dry season (EDS), late dry season (LDS) and total emissions for 10 West African savanna countries (>600 mm rainfall per year) generating greater than 50,000 tCO_2_-e yr^−^^1^ LDS (data originates from Lipsett-Moore et al. 2018).

No	Country	Mean EDS tCO_2_-e	%	Mean LDS tCO_2_-e	%	Total tCO_2_-e
1	Burkina Faso	566,833	81	136,732	19	703,566
2	Senegal	738,830	77	218,938	23	957,768
3	Benin	1,023,387	75	341,300	25	1,364,687
4	Togo	476,413	63	278,359	37	754,772
5	Ghana	1,566,081	60	1,055,595	40	2,621,676
6	Côte d’Ivoire	1,408,080	50	1,402,683	50	2,810,763
**7**	**Mali**	**700,574**	**41**	**1,021,034**	**59**	**1,721,608**
8	Nigeria	381,538	11	3,153,920	89	3,535,458
9	Sierra Leone	45,213	8	526,922	92	572,135
10	Guinea-Bissau	24,875	7	344,745	93	369,620
	Total	**6,931,824**	45	**8,480,229**	55	**15,412,053**

## The West African patch-mosaic burning regime

Laris also notes that people in West Africa overwhelmingly set early dry season (EDS) fires. This is true for Burkina Faso, Senegal, Benin, Togo, Ghana, which all have an early burning pattern (See Table 1). However, this is not the case for Nigeria, Sierra Leone and Guinea-Bissau, which have most emissions in the late dry season (LDS) (see Table 1). Also, if we sum the total EDS and LDS emissions for West African Countries, then 45% of emissions occur in the EDS and 55% in the late dry season (see Table 1). The total West African contribution is around 8% of the total African savanna emissions—a relatively small contributor.

We haven’t suggested that the early burning practise would work for all of West Africa, but the evidence suggests that it would work for Nigeria, Sierra Leone and Guinea-Bissau (see Table 1). We agree, many of the West African countries have significant EDS burning patterns like Burkina Faso, Senegal, Benin, Togo and Ghana and would not benefit from the approach. However, for those countries with significant EDS burning that still have significant LDS emissions as well, such as Mali and Côte d’Ivoire, there may be some opportunity for further emissions reductions through improved fire management practices as presented in our paper^3^.

Laris^1^ also points out that the same EDS regime proposed is one that was developed by indigenous people and that it has been applied by Africans for centuries. The same is true for Australia, but colonial occupation altered that, as it has in some areas of Africa. A new incentive in the form of carbon payments for early burning in Australia has empowered local indigenous people to reconnect to their traditional lands and fulfil their cultural obligations and a diversity burning practices^14^.

## The fire dichotomy

We chose the driest month as the simplest repeatable framework. We recognize that there are many other approaches (e.g. Chandler Burning Indices, Vegetation Dryness Indices and many others as suggested). For example, the approved methodology in Australia uses set dates to define EDS (1 January–31 July) and LDS (1 August–30 December) fires^2^. This simple approach works for most of northern Australia but is problematic in Cape York which is wetter for longer. Consequently, under the new methodologies, there are new approaches for defining EDS and LDS better aligned to regional differences in seasonality^12^.

EDS fires in northern Australia are equivalent to the (MDS) described by Laris^10^. Fires are generally set in June and July when the grasses have cured, conditions are cool and when effective back burning can occur to produce cool and controlled fires. Ultimately, we agree with Laris that it will be important to refine the general approach we describe with the most locally relevant characterization of fire seasonality.

Finally, EDS fire management is not a panacea for Africa as stated in our paper, but there are countries, people and environments that will benefit from EDS fire management driven by Indigenous landholders and the carbon, cultural, social, economic and environmental benefits that can be generated^14^. This approach for fire management by indigenous communities has proven successful with respect to multiple objectives in Australia and given that savanna fire management originated in Africa, it is only fair that African communities benefit too.

## Data Availability

All data used in this article are publicly available and the data analysed and supporting the findings of this study are available from the corresponding author on reasonable request. Primary datasets used in the analyses include: GPCC 0.5°, long-term (1981–2010) monthly mean precipitation dataset (https://www.esrl.noaa.gov/psd/data/gridded/data.gpcc.html); GFED4 (https://daac.ornl.gov/VEGETATION/guides/fire_emissions_v4_R1.html) and WDPA (https://www.iucn.org/theme/protected-areas/our-work/world-database-protected-areas).

## References

[1] Laris, P. On the problems and promises of savanna fire regime change. *Nat. Communications.* (2021). 10.1038/s41467-021-25141-1 PMC8361073 34385438

[2] Commonwealth of Australia. Carbon credits (carbon farming initiative—emissions abatement through savanna fire management) methodology determination. https://www.legislation.gov.au/Details/F2015L00344 (2015).

[3] Lipsett-Moore, G. J., Wolff, N. H. & Game, E. T. Emissions mitigation opportunities for savanna countries from early dry season fire management. *Nature Communications*, **9**, 2247 (2018) 10.1038/s41467-018-04687-7 PMC5993717 29884858

[4] Intergovernmental Panel on Climate Change (IPCC). Guidelines for National Greenhouse Gas Inventories. Vol. 4, Agriculture, Forestry and Other Land Use (2006).

[5] Hoffa EA (1999). Seasonality of carbon emissions from biomass burning in a Zambian savanna. J. Geophys. Res..

[6] Korontzi S, Justice CO, Scholes RJ (2003). Influence of timing and spatial extent of savanna fires in southern Africa on atmospheric emissions. J. Arid Environ..

[7] Korontzi S (2005). Seasonal patterns in biomass burning emissions from southern African vegetation fires for the year 2000. Glob. Change Biol..

[8] Meyer CP (2012). Direct measurements of the seasonality of emission factors from savanna fires in northern Australia. J. Geophys. Res..

[9] Russell-Smith, J. et al. Opportunities and challenges for savanna burning emissions abatement in southern Africa *J. Environ. Manage.* **288**, 112414 (2021). 10.1016/j.jenvman.2021.112414 33831642

[10] Laris, P., Jacobs, R., Koné, M., Dembélé, F., & Rodrigue, C. M. Determinants of fire intensity in working landscapes of an African savanna. *Fire Ecology* 16:27 (2020).

[11] Cook GD, Meyer CP, Muepu M, Liedloff AC (2016). Dead organic matter and the dynamics of carbon and greenhouse gas emissions in frequently burnt savannas. Int. J. Wildland Fire.

[12] Commonwealth of Australia. Carbon Credits (carbon farming initiative—savanna fire management—sequestration and emissions avoidance) methodology determination. https://www.legislation.gov.au/Series/F2018L00562 (2018).

[13] Russell-Smith, J. Key research to assist the development of Emissions Reduction Fund carbon sequestration methods for savanna fire management in Northern Australia Darwin Centre for Bushfire Research, Charles Darwin University (Meat and Livestock Australia/Australian Government). https://www.mla.com.au/research-and-development/search-rd-reports/final-report-details/Key-research-to-assist-the-development-of-Emissions-Reduction-Fund-carbon-sequestration-methods-for-savanna-fire-management-in-Northern-Australia/4410 (2020).

[14] Ansell, J. et al. Contemporary Aboriginal savanna burning projects in Arnhem Land: a regional description and analysis of the fire management aspirations of Traditional Owners. *Int. J. Wildland Fire* https://www.publish.csiro.au/wf/WF18152 (2019).

